# Usability of a Consumer Health Informatics Tool Following Completion of a Clinical Trial: Focus Group Study

**DOI:** 10.2196/17708

**Published:** 2020-06-15

**Authors:** Hwayoung Cho, Tiffany Porras, Gabriella Flynn, Rebecca Schnall

**Affiliations:** 1 College of Nursing University of Florida Gainesville, FL United States; 2 School of Nursing Columbia University New York, NY United States

**Keywords:** consumer health informatics tool, mobile Health, mobile apps, clinical trial, symptom care, self-management, HIV-associated nonAIDS (HANA), HANA conditions, HIV/AIDS

## Abstract

**Background:**

Mobile health (mHealth) apps have the potential to be effective tools for encouraging patients with chronic diseases to self-manage their health. The success of mHealth apps is related to technology acceptance and its subsequent use by intended consumers. Therefore, it is essential to gain insights from consumers’ perspectives about their use of mHealth apps in daily life.

**Objective:**

The purpose of this work was to understand consumers’ perspectives on use of a self-management app following completion of a clinical trial that tested the efficacy of the app for improving health outcomes.

**Methods:**

We conducted five focus groups with paricipants of a clinical trial (NCT03182738) who were randomized to use the video information provider (VIP) for HIV-associated nonAIDS (HANA) conditions app (VIP-HANA) or an attention control app. Thematic analysis was conducted, and the themes were organized according to the two key constructs of the technology acceptance model framework: perceived usefulness and perceived ease of use.

**Results:**

Thirty-nine people living with HIV (20 from the intervention group and 19 from the control group) participated in the focus group sessions. Of the eight themes identified from focus group data, the five themes related to perceived usefulness were: (1) self-monitoring HIV-related symptoms of HANA conditions, (2) enhanced relationship with clinical providers, (3) improvement in physical and emotional health, (4) long-term impact of self-care strategies on improvement in symptoms of HANA conditions, and (5) inspired lifestyle changes to manage symptoms. The three themes related to perceived ease of use were: (1) easy to navigate, (2) avatar personalization, and (3) privacy/confidentiality maintained even when changing the location of app use.

**Conclusions:**

Perceived ease of use was similar in both study groups but perceived usefulness differed between study groups. Participants in both study groups found the VIP-HANA app to be useful in monitoring their symptoms and enhancing communication with their clinical care providers. However, only intervention group participants perceived the app to be useful in improving overall health and long-term symptom management. Findings from this study highlight factors that are essential to ensure the usefulness of self-management apps and facilitate sustained use of mHealth apps for people living with chronic illnesses.

## Introduction

With the widespread proliferation of smartphones, the use of mobile technology has rapidly increased in health care [1,2]. Consumer health informatics tools such as mobile health (mHealth) apps have the potential to be effective tools to encourage patients with chronic diseases to self-manage their health [3-7]. The video information provider (VIP) for HIV-associated nonAIDS (HANA) conditions app (VIP-HANA) is an mHealth app that was developed to help people living with HIV self-manage their symptoms by providing self-care strategies [8]. Development of the VIP-HANA app is described elsewhere [9]. In short, the VIP-HANA app is comprised of 728 self-care strategies for the following 28 HANA condition-related symptoms: fatigue, depression, muscle aches, difficulty falling asleep, anxiety, difficulty staying asleep, difficulty remembering, neuropathy, difficulty concentrating, decreased sex drive, diarrhea, maintaining an erection, shortness of breath, constipation, dry mouth, clumsiness, weight loss, dizziness, heartburn, dry eyes, changes in appetite, ringing in ear, cough, nausea/vomiting, fever, difficulty with urination, speech difficulties, and pain during sex [10]. The 728 self-care strategies were tailored based on the symptom, gender, race/ethnicity, and HANA condition of each user. An avatar guides users through the app. Patients then receive self-care strategies with animated videos for ameliorating their symptoms. Users review their symptom experience over time with symptom reports, set a weekly email reminder to complete sessions in the app, and email/download the history of their reported symptoms and suggested self-care strategies [8].

Two versions of the app were created to assess the efficacy of the VIP-HANA app in improving the symptom burden in a 6-month randomized controlled trial (RCT) (NCT03182738) that included 100 people living with HIV with HANA conditions (50 randomly assigned to the intervention group, 50 assigned to the control group). All participants were asked to log in at least once per week. Both study groups received weekly symptom assessment questions through the VIP-HANA app, yet only the intervention group participants were provided with the self-care strategies. Multimedia Appendix 1 presents a comparison of the two versions of the VIP-HANA app features. Participants in both groups were given US $5 per week for their time and data costs associated with completing the weekly symptom assessments.

According to a recent US national survey of mHealth app use, approximately half (46%) of the respondents reported downloading a health app but were no longer using it or had uninstalled it [11]. Given that the success of mHealth apps is related to the acceptance of technology and its subsequent use by intended consumers, there is a need to gain in-depth insights from consumers’ perspectives about use of mHealth apps in their daily life. Accordingly, the purpose of this study was to understand consumers’ perspectives on mHealth use (VIP-HANA app) following an RCT of an HIV symptom self-management intervention for people living with HANA conditions.

## Methods

### Sampling and Recruitment

This study was a follow up of a 6-month trial including 100 people living with HIV with HANA conditions (50 randomly assigned to the intervention group, 50 to the control group). We used a convenience sampling approach to recruit a subsample of our study participants to participate in one of five focus group sessions followed by completion of the study trial; the focus group sessions took place once per month [12-14].

### Procedures

The Institutional Review Board of Columbia University Medical Center reviewed and approved all research activities. We conducted three focus group sessions with intervention group participants (N=20) and two focus groups sessions with control group participants (N=19). Prior to the focus group sessions, all participants were provided an explanation of the study procedures and completed a signed consent form. Using open-ended questions via a semistructured focus group guide designed based on the app’s features/functionality (Textbox 1), a moderator (a faculty member at Columbia University School of Nursing) facilitated the focus group sessions, and participants were encouraged to discuss issues regarding their perceptions related to use of the VIP-HANA app (intervention/control group versions). All focus group sessions were audio-recorded. Data collection continued until saturation of themes was reached. Participants were compensated with US $30 for their time.

**Focus group guide based on 6-month experience of using the video information provider for HIV-associated nonAIDS conditions (VIP-HANA) app.**

**Experience Using VIP-HANA**

- Please describe your experience using the VIP-HANA app in social settings.
  Probe: where and when did you use it most (house/workplace/clinic/café; after breakfast/when commuting, etc) and why?
- How did this fit into your lifestyle and schedule?
- Please describe your experience navigating the app pages.
  Probe: basic structure of the app
- After your first app use, how easy/difficult was it for you in follow-up uses?
- What are your thoughts about the design of the VIP-HANA app?
  Probe: main logo/avatars; font/color; progress bar; Remember Me (save ID/password); Log-In Help; Continue/Skip button
- Please describe your experience with any technical issues.
  Probe: Log-In, crash, Back button, Continue/Skip button, How Our App Works, reminder/email, error message

(Intervention group only)

- What are your thoughts about the videos displayed in the VIP-HANA app?
  Probe: watching videos vs reading the content; videos with sounds, the type of sounds; effect of data plan on watching the videos
- What was your experience using the app to review your symptoms and self-care strategies (=Your History)?

**Impact of VIP-HANA**

- How did this app help you gain information about relieving your symptoms?
- How do you think that your symptoms changed after using the VIP-HANA app for 6 months?
- How confident are you in your ability to self-manage your symptoms?
- What are some of the ways that your health might benefit through the use of VIP-HANA?
  Probe: changes of current personal, professional, or health care provider relationships
- Please describe how the app did/can change your quality of life.

### Data Analysis

**Figure 1:**
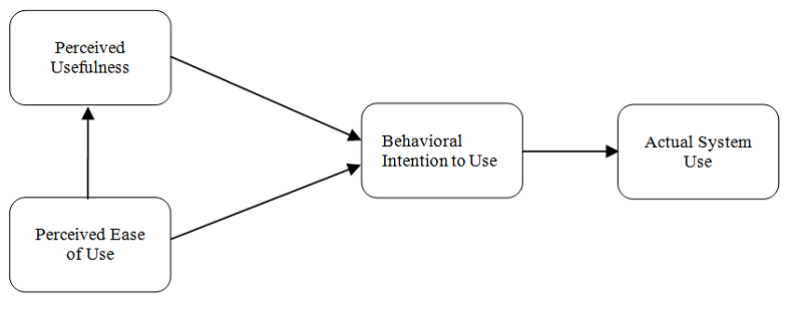
Technology acceptance model (TAM).

The technology acceptance model (TAM) was used as a guide for the data analysis [15]. The TAM includes the following two key constructs: perceived usefulness and perceived ease of use. Perceived usefulness represents the degree to which an individual believes that using a particular system would enhance one’s job performance, whereas perceived ease of use signifies the degree to which an individual believes that using a particular system would be free of effort. The TAM suggests that these two constructs of user acceptance influence the behavioral intention to use a system and predict the extent of adoption of the system (Figure 1).

Audio recordings from the focus groups were transcribed. Thematic analysis was used to explore significant themes with similar patterns across focus group sessions. Research team members (HC, TP, and GF) independently reviewed the transcripts at least twice, and two research team members (TP and GF) generated a set of codes through a combination of inductive and deductive content analysis [16-18]. A codebook was developed using a Microsoft Excel spreadsheet, and free text excerpted from the transcripts was entered into the codebook followed by each of the themes. Coding discordance between TP and GF was reconciled by a third researcher (HC). Guided by the TAM framework (Figure 1) [15], each theme in the codebook was categorized into the two constructs of perceived usefulness and perceived ease of use.

## Results

### Sample

A total of 39 people who completed the 6-month trial participated in the five focus group sessions. The mean age of the study participants was 55 years (SD 10.70). Additional characteristics of the study sample are presented in Table 1.

**Table 1:** Characteristics of the study participants (N=39).

Characteristic		All participants (N=39)	Intervention (N=20)	Control (N=19)
**Gender, n (%)**				
	Male	22 (56)	12 (60)	10 (53)
	Female	15 (38)	7 (35)	8 (42)
	Transgender	1 (3)	0 (0)	1 (5)
	Genderqueer	1 (3)	1 (5)	0 (0)
**Sex at birth, n (%)**				
	Male	24 (62)	13 (65)	11 (58)
	Female	15 (38)	7 (35)	8 (42)
**Race, n (%)**				
	African American/Black	27 (69)	13 (65)	14 (74)
	Other	8 (21)	4 (20)	4 (21)
	White	4 (10)	3 (15)	1 (5)
Hispanic/Latino ethnicity, n (%)		13 (33)	6 (30)	7 (37)
**Marital status, n (%)**				
	Single	22 (56)	12 (60)	10 (53)
	In a relationship	13 (33)	6 (30)	7 (37)
	Legally married/domestic partnership	2 (5)	1 (5)	1 (5)
	Widowed	1 (3)	0 (0)	1 (5)
	Divorced	1 (3)	1 (5)	0 (0)
**Education level, n (%)**				
	High school diploma or equivalent	14 (36)	6 (30)	8 (42)
	Some college	10 (26)	6 (30)	4 (21)
	Some high school, no diploma	6 (15)	3 (15)	3 (16)
	Associate or technical degree	4 (10)	1 (5)	3 (16)
	Bachelor/college degree	2 (5)	1 (5)	1 (5)
	Professional or graduate degree	2 (5)	2 (10)	0 (0)
	None	1 (3)	1 (5)	0(0)
**Current employment status, n (%)**				
	Disabled	16 (41)	8 (40)	8 (42)
	Unemployed	12 (31)	6 (30)	6 (31)
	Retired	5 (13)	3 (15)	2 (11)
	Working part time	4 (10)	3 (15)	1 (5)
	Retired and other	2 (5)	0 (0)	2 (11)
	Working full time	0 (0)	0 (0)	0 (0)
**Annual income, n (%)**				
	Less than US $10,000	18 (46)	11 (55)	7 (37)
	US $10,000-$19,000	8 (21)	3 (15)	5 (26)
	US $20,000-$39,999	7 (17)	4 (20)	3 (16)
	Don’t know	5 (13)	1 (5)	4 (21)
	US $40,000-$59,000	1 (3)	1 (5)	0 (0)
**Health insurance provider, n (%)**				
	Public (Medicare, Medicaid, Ryan White)	32 (82)	16 (80)	16 (84)
	Other (eg, ADAP^a^, Veteran’s Association, multiple)	6 (15)	3 (15)	3 (16)
	None	1 (3)	1 (5)	0 (0)
	Private (through employer)	0 (0)	0 (0)	0 (0)

^a^ADAP: AIDS drug assistance program.

### Main Themes

A total of eight themes related to use of the VIP-HANA app were identified. The themes were organized by the two key constructs of the TAM framework [15], including five themes related to perceived usefulness and three themes related to perceived ease of use. Two themes to enhance the acceptance of the app were additionally identified.

#### Perceived Usefulness

The five themes related to perceived usefulness were: (1) self-monitoring HIV-related symptoms of HANA conditions, (2) enhanced relationship with clinical providers, (3) improvement in physical and emotional health, (4) long-term impact of self-care strategies on improvement in symptoms of HANA conditions, and (5) inspired lifestyle changes to manage symptoms.

##### Self-Monitoring HIV-Related Symptoms of HANA Conditions

Participants in both the intervention and control groups felt that the app was helpful for improving self-monitoring of their symptoms. A control group participant expressed that the app helped them realize the frequency of certain symptoms they were having, by stating:

I found that it really kept me in touch with my symptoms. And a lot of things were happening that I really didn’t realize until I started using the app.

FG2

An intervention group participant mentioned:

The app shows you certain things that you can have, and what you can do. And you work with that. And it’s been great for me. I was getting more confident with tracking and managing my symptoms.

FG3

Moreover, intervention group participants described enjoyment in tracking their HANA conditions:

So for those of us that do have secondary illnesses such as diabetes, asthma, high blood pressure, and so forth and so on, the app was a great thing, and not just helping us navigate the system and our bodies as far as HIV is concerned, but helped us in those other issues as well.

FG1

And she said the app is wonderful… for diabetes, hepatitis C, and for the different harm reduction. We know what symptoms I have.

FG3

##### Enhanced Relationship With Clinical Providers

Participants in both the intervention and control groups stated that they showed the app to their doctors/clinical therapists to guide their conversations and share their symptom reports, which empowered them to better communicate with their clinical providers and enrich their relationship. An intervention group participant described:

The changes that I made were, the app was very useful in that, in conjunction with my doctor. Because I also showed this to my doctor… And they all agreed that it’s a wonderful thing.

FG1

A control group participant stated:

I liked the app because it was times I wanted to ask my doctor questions, but I didn’t. But by doing this here app, it made me take a pen and paper and write down what I want to say to her next time I see her.

FG2

##### Improvement in Physical and Emotional Health

Participants in the intervention group noted improvements in their health as a result of app use. Participants commented that the app helped them work through their emotional issues. An intervention group participant stated:

It is not just HIV health. It is diabetes, asthma, high blood pressure… and so on… Not just your physical and your medications and stuff like that. But things that you can do to make yourself happier, better, more social.

FG1

##### Long-Term Impact of Self-Care Strategies on Improvement in Symptoms of HANA Conditions

Intervention group participants stated that they continued to use the self-care strategies for their symptoms even after the study was over. They considered that the self-care strategies resulted in a lasting improvement of their symptoms and therefore they continued to use them. An intervention group participant mentioned:

When I go through stress, I get those migraine headaches. In the app, it tells you to relax, turn off the TV. Turn off everything and just be calm in a dark room. And that still helps me. I am still doing that.

FG1

##### Inspired Lifestyle Changes to Manage Symptoms

Participants in the control group noted that the symptom questions on the VIP-HANA app helped them to think about their recent symptoms and inspired lifestyle changes. For example, some control group participants stated:

I remember one time, I was really depressed that week. And I put down real severe depression. And it made me think... So I’m trying to figure out why am I depressed? What made me depressed this week? What should I do? What changes?

FG2

But when you're looking at, when you're pressing this button several times every, several times a month, with the same thing, it reminds you. You need to really get serious about what you've been thinking about all this time.

FG2

#### Perceived Ease of Use

The three major themes related to perceived ease of use were: (1) easy to navigate, (2) avatar personalization, and (3) privacy/confidentiality maintained even when changing the location of app use.

##### Easy to Navigate

Participants in both the intervention and control groups found that the VIP-HANA app was easy to navigate, and they were able to use the app without assistance and did not experience technical issues. An intervention group participant stated:

Nothing hard at all. I am not very good to the tech… but this app, easy to get to start… easy to go to the next… very easy to move forward.

FG3

A control group participant described:

Yeah. The app I thought was pretty easy to navigate through. And the questions were basically always the same. I didn’t need any help although I am not good at technology.

FG2

##### Avatar Personalization

Participants in the intervention and control groups noted the usefulness of an avatar to guide them through the VIP-HANA app and help them navigate through the app. They also noted that the avatar promoted their engagement with the app. Some participants in the intervention group mentioned:

I feel like I connected to it more by choosing the one that best looked like me or was fitted to me.

FG1

When you see it in the app, where you’re actually seeing the cartoon character [avatar], get up and going. It encourages you that you can get up and go. I think it was very practical.

FG5

Some participants in the control group stated:

I think when you look at it [avatar], it’s more personal. It’s about you. And I think that it’s good for your psyche to realize that you’ve got to really take care of yourself.

FG2

The app I thought was pretty easy to navigate through because of the avatar. The avatar looks like me… myself!

FG4

I was going to mention that the whole thing is laid out pretty attractively. The little avatar made it, to me, it made it almost like a game.

FG2

##### Privacy/Confidentiality Maintained Even When Changing Location of App Use

Participants in both the intervention and control groups felt comfortable using the app in a variety of locations ranging from their home to doctor’s offices and in public spaces (ie, everywhere). They thought that the app was very discrete and did not disclose their HIV status to others if they could see the app on the screen. Several participants in the intervention group described this aspect:

To me, I feel like it was enough discretion in the app that if I wanted to explain it to somebody, because I was okay pulling out anywhere… everywhere…

FG1

I feel that that was really good, in the sense of you can do it really anywhere.

FG5

Some participants in the control group stated:

One of the good things about the program, it doesn’t mention anything related to HIV. The reason… If someone want to get into your phone, nobody can access personal information related to the app.

FG2

I was happy because I can use it anytime and anywhere; at work, on the train.

FG4

#### Additional Comments to Enhance App Acceptance

##### Need for Updates of Self-Care Strategies and Videos

Participants in the intervention group expressed their desire for the app to include more self-care strategies and realistic videos. Participants felt that the app questions (symptoms) and suggestions (self-care strategies) were particularly useful for people living with HIV who were newly diagnosed with HIV/AIDS. They thought that the animated videos showing the suggestions were too simple or not serious enough in some cases. Some participants in the intervention group described:

When I look at the study you have to say that it’s really geared more to like an HIV 101 plan where it’s just basic stuff… For a person that’s newly diagnosed and don’t know a lot of things about HIV, I think it’s the best thing for them.

FG1

Some of it [animated videos] was unrealistic. If you don’t have the HIV or the pain or things that we actually experience as patients, you really couldn’t understand how serious sometimes it is. It felt to me like they downplayed our experiences with those videos.

FG3

##### Request for Additional Functionality of Symptom Reports

Participants in the control group wanted the app to have an additional functionality to track their symptom reports so that they do not have to remember their weekly answers to the symptom questions (eg, what symptoms they had), as the app feature of symptom reports was limited for the intervention group participants only in this study. For example, a control group participant stated:

I don’t remember there being an ability to be able to say, to run a report, so to speak, and say; between January and March, I had dry eyes this many times during the month... if I can print something out or if I can open it on the phone and show it to the doctor like that, that would be a nice addition to what you already have.

FG2

## Discussion

### Principal Findings

With the exponential increase of mobile technology use in health care, mHealth apps can be a promising consumer health informatics tool for health behavior change and self-management. Although existing evidence supports the use of mHealth apps to improve health outcomes in people with chronic diseases [3-7], there has been limited assessment of consumers’ experience following their use of these apps during a clinical trial. Given the current landscape of discontinuation of mHealth apps use [11], it is imperative to understand how consumers are interacting with the apps in their daily life in an effort to accelerate acceptance of the apps by the consumers as well as ensure their subsequent use. To better understand consumers’ use of a self-management app, we included both intervention and control group participants following completion of an RCT that tested the efficacy of the app for improving health outcomes. We captured valuable perspectives of both study group participants on their app use using focus groups guided by a theory-driven framework. This approach facilitated gaining an in-depth understanding of the key factors that would have a critical influence on adoption of the app [15], which is a significant strength of our study.

Participants in both the intervention and control groups perceived the VIP-HANA app to be useful for monitoring their symptoms, and the app enhanced their relationship with their clinical providers. Although self-care strategies of existing symptoms were not provided to the control group participants, all participants described the need to record, track, and share their symptom status with their providers. Participants described that use of the symptom reports would expedite opportunities of health communication and enhance their relationship with their clinical providers. Current research supports that interpersonal communication between HIV patients and their health care provider improves medication adherence and long-term care in HIV, highlighting the importance of patient-provider communication for ensuring that patients remain in the continuum of HIV care [19-21].

Although all three themes related to perceived ease of use were identified in both study groups, only the intervention group participants described the VIP-HANA app as being useful for enhancing their physical and emotional health and supporting long-term symptom management. Existing evidence suggests that perceived usefulness is a stronger factor compared with perceived ease of use for influencing technology adoption, and perceived usefulness has a positive influence on overall use of an app [22,23].

mHealth apps should be visually appealing to consumers [24], but more fundamentally should contain evidence-based health information that consumers can use to improve their health. Participants considered that the app could help improve their health and manage symptoms through the use of self-care strategies. The VIP-HANA app was developed by employing evidence from patient-centered outcomes research studies [25], and the HIV symptom self-care strategies provided within the app were tailored based on symptoms, gender, race/ethnicity, and HANA condition [26,27]. To our knowledge, no mHealth intervention offers health information on how to ameliorate HIV symptoms specifically for people living with HIV with HANA conditions; the VIP-HANA app therefore represents a first step in the development and dissemination of a consumer health informatics tool that incorporates evidence-based self-care strategies.

One of the major themes related to perceived ease of use in this study was avatar personalization. Within both group versions of the VIP-HANA app, participants were guided by an avatar through a series of questions ascertaining the nature and severity of their symptoms. The avatar then recommended self-care strategies for addressing the intervention group participants’ symptoms. Even though the avatar in the control group version of the app did not provide any suggestions after the symptom assessment questions, participants in both groups felt that they connected to the app simply by selecting an avatar that most looked like them. Current research suggests that HIV patients feel more comfortable receiving HIV-related questions/answers from avatars compared to receiving health information directly from clinical providers, and users tend to choose an avatar that matches their own ethnicity [28,29]. Given that avatars can encourage user interaction with health-related information, developers of consumer health informatics tools should consider the potential benefits of inclusion of customizable avatars.

Privacy/confidentiality was a factor related to perceived ease of use. Our participants noted that the information (eg, data of participants’ experienced symptoms) displayed on an app was secure because the app was installed on their own smartphone, and the app’s design did not disclose their HIV status; thus, they felt comfortable using the app everywhere. In designing the app, privacy and confidentiality were strong considerations since people living with HIV in our formative work emphasized their importance [30] and social stigma associated with HIV persists [31]. Findings from our study emphasize the value of using mobile-based interventions, particularly for people living with HIV, and the importance of a user-centered design process with inclusion of users in terms of ensuring data security/protection.

Control group participants highlighted the need for inclusion of symptom reports in order to share their information with clinical providers without needing to memorize their symptom change/progress during the past 6 months. This functionality was only available for intervention group participants. Visual communication displayed on a summary on the app may improve patient comprehension of health information [32,33]. Findings from this work showed that graphical symptom reports enhanced the functionality of the app, which is an important implication for the future development of mHealth apps.

### Limitations

There are some limitations to the generalizability of our results. Study participants who participated in the focus groups might have been those who were most likely to be engaged with and used the app who had been retained in the 6-month trial. More than half of our study participants were men, African American/Black, those who had completed some high school or less, and those who reported an annual income of less than US $20,000. Therefore, the Hawthorne effect might influence the study findings of focus groups [34]. We designed the semistructured focus group guide based on our app’s features and functionality, and used open-ended questions to minimize/eliminate concern about the Hawthorne effect.

### Conclusions

Understanding factors influencing the use of patient-centered tools is critical. This study highlights participants’ experiences, attitudes, and perceptions of their use of a behavioral intervention evaluated in a clinical trial. Participants were able to integrate the app into their daily routine, and used the app to support, track, monitor, and self-manage HIV symptoms related to HANA conditions. Although perceived ease of use was similar in both study groups, perceived usefulness differed between the groups. Participants in both groups found the VIP-HANA app to be useful in monitoring their symptoms of HANA conditions and enhancing communication with their clinical providers over time, but only the intervention group participants perceived the app to be useful for improving overall health and long-term symptom management in the HIV care continuum. Findings from this study highlight factors that are essential to ensure the usefulness of self-management apps to promote sustained use of the apps for people living with chronic illnesses.

## Appendix

Multimedia Appendix 1 Comparison of the VIP-HANA app features in the intervention and control group versions.

## Abbreviations

HANA: HIV-associated nonAIDS
mHealth: mobile health
RCT: randomized controlled trial
TAM: technology acceptance model
VIP-HANA: video information provider for HIV-associated nonAIDS conditions
