# Supplementary material for: Usability of a Consumer Health Informatics Tool Following Completion of a Clinical Trial: Focus Group Study
Source: J Med Internet Res. 2020 Jun 15;22(6):e17708. doi: 10.2196/17708 (PMC7325002; doi:10.2196/17708)
Supplement: Multimedia Appendix 1 [file jmir_v22i6e17708_app1.docx]

| VIP-HANA app features | Intervention group version | Control group version |
| --- | --- | --- |
| **Home screen**  **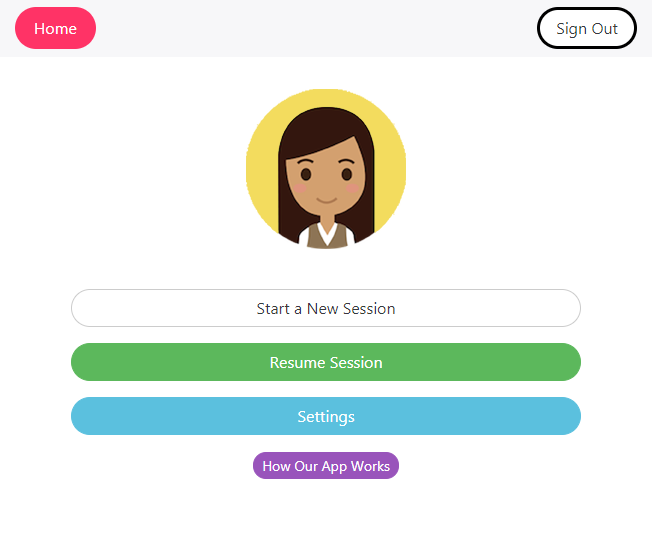** | o | o |
| **Avatar selection**  **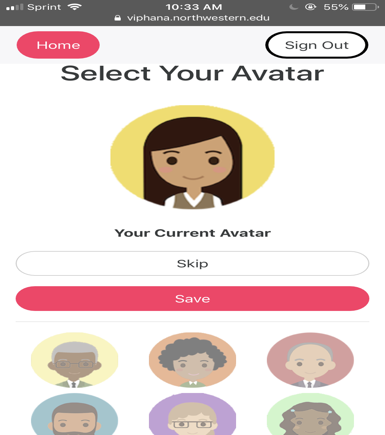** | o | o |
| **Weekly email reminder**  **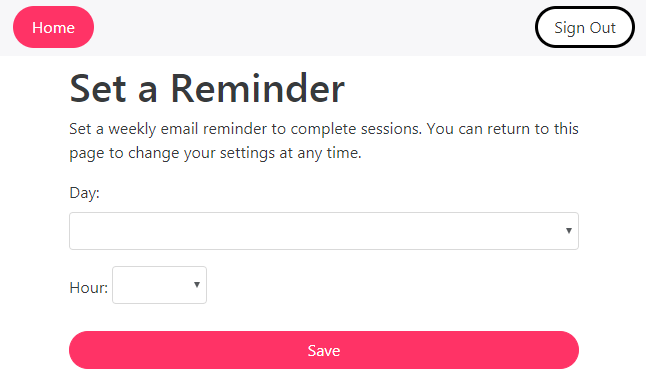** | o | o |
| **Weekly symptom assessment**  **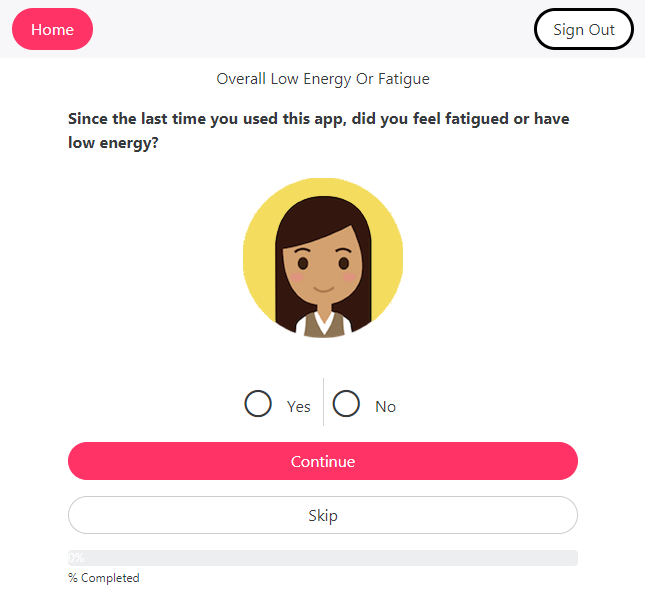** | o | o |
| **Self-care strategies/videos for the reported symptom**  **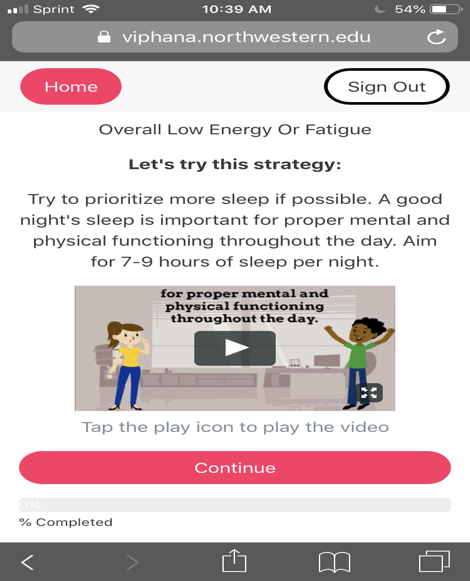** | o | ^x |
| **Email/download of the reported symptoms/suggested self-care strategies**  **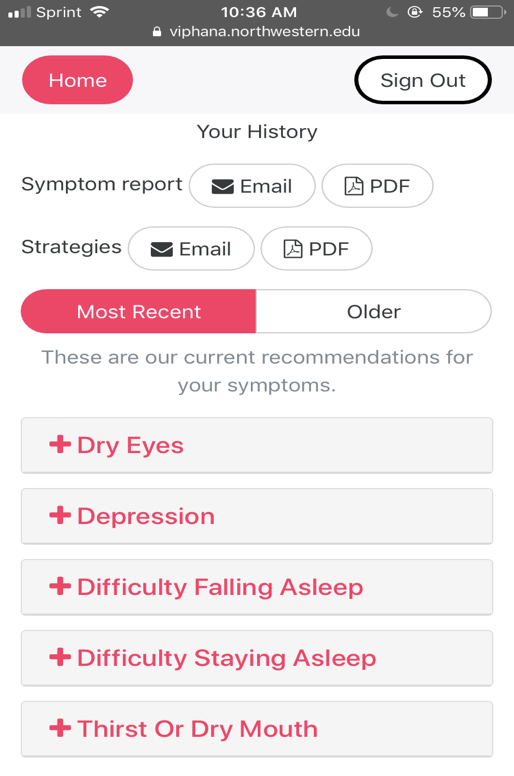** | o | x |
| **Review of symptom report/strategies: symptom reports**  **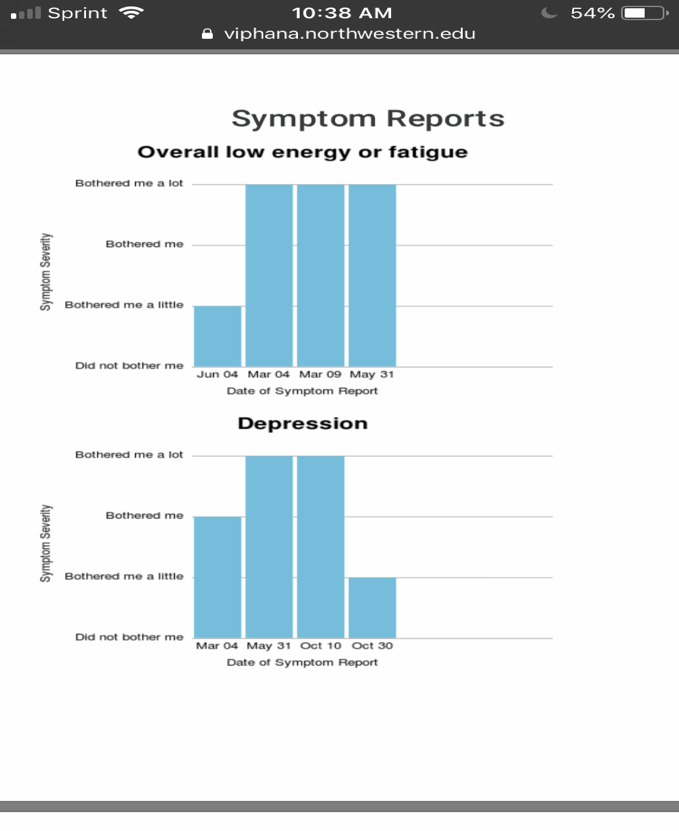** | o | x |
| *o (provided through the VIP-HANA app); ^x (not provided through the VIP-HANA app) | | |
